# Cost-effectiveness of alternative strategies for use of 13-valent pneumococcal conjugate vaccine (PCV13) in Canadian adults

**DOI:** 10.17269/s41997-018-0050-9

**Published:** 2018-05-09

**Authors:** Mark Atwood, Linda Beausoleil, Marie-Claude Breton, Craig Laferriere, Reiko Sato, Derek Weycker

**Affiliations:** 1 Policy Analysis Inc. (PAI), Four Davis Court, Brookline, MA 02445 USA; 2 Pfizer Canada Inc., Kirkland, Quebec, Canada; 3 Pfizer Inc., Collegeville, PA USA

**Keywords:** Cost-effectiveness analysis, Pneumococcal infection, Pneumococcal pneumonia, Pneumococcal vaccines, 13-valent pneumococcal vaccine, Analyse coût-efficacité, Infection à pneumocoque, Pneumonie à pneumocoque, Vaccins contre le pneumocoque, Vaccin 13-valent contre le pneumocoque

## Abstract

**Objectives:**

The Canadian National Advisory Committee on Immunization (NACI) recommends use of 13-valent pneumococcal conjugate vaccine and 23-valent pneumococcal polysaccharide vaccine in a sequential schedule (PCV13 → PPV23) among adults aged ≥ 65 years and those aged ≥ 18 years who are immunocompromised. In light of recent PCV13 efficacy data from the Community-Acquired Pneumonia Immunization Trial in Adults (CAPiTA), and new sero-epidemiology data on community-acquired pneumonia (CAP), we examined the economic implications of funding an expanded adult pneumococcal immunization program in Canada.

**Methods:**

A microsimulation model depicting expected lifetime risks, consequences, and costs of invasive pneumococcal disease (IPD) and CAP was developed. PPV23 effectiveness was based on published literature, and PCV13 effectiveness was based on CAPiTA; all other model parameters were based on published data or secondary sources. Herd effects from the PCV13 pediatric program were considered. Outcomes and costs were evaluated assuming use of PPV23 alone, and alternatively, use of PCV13 → PPV23 among (1) all adults aged ≥ 65 years (*n* = 5.4 M) and (2) immunocompromised and high-risk adults aged ≥ 65 years (*n* = 3.0 M).

**Results:**

For population no. 1, PCV13 → PPV23 reduced IPD cases by 1100, CAP cases by 7000, and disease costs by $135.8M; vaccination costs increased by $254.3M, and cost per QALY gained was $35,484. For population no. 2, PCV13 → PPV23 reduced IPD cases by 900, CAP cases by 6000, and disease costs by $120.3M; vaccination costs increased by $149.8M, and cost per QALY gained was $10,728.

**Conclusion:**

Expanding use of PCV13 → PPV23 by funding PCV13 among Canadian adults aged ≥ 65 would be a cost-effective use of healthcare resources.

**Electronic supplementary material:**

The online version of this article (10.17269/s41997-018-0050-9) contains supplementary material, which is available to authorized users.

## Résumé

**Objectifs:**

Le Comité consultatif national de l’immunisation (CCNI) du Canada recommande l’administration du vaccin 13-valent conjugué contre le pneumocoque et du vaccin polysaccharidique 23-valent contre le pneumocoque selon un schéma séquentiel (PNEU-C-13 → PNEU-P-23) aux adultes de ≥65 ans et aux personnes immunodéprimées de ≥18 ans. À la lumière des données récentes sur l’efficacité du PNEU-C-13 tirées de l’étude CAPiTA (Essai d’immunisation active pour la prévention de la pneumonie communautaire chez l’adulte) et de nouvelles données séro-épidémiologiques sur les pneumonies d’origine communautaire (POC), nous avons examiné les répercussions économiques du financement d’un programme élargi de vaccination des adultes contre le pneumocoque au Canada.

**Méthode:**

Nous avons élaboré un modèle de microsimulation dépeignant les risques à vie escomptés, les conséquences et les coûts des pneumococcies invasives (PI) et des POC. L’efficacité du PNEU-P-23 a été déterminée en fonction de la documentation publiée, et celle du PNEU-C-13, d’après l’étude CAPiTA; les autres paramètres du modèle proviennent de données publiées ou de sources secondaires. L’immunité collective conférée par le programme d’administration pédiatrique du PNEU-C-13 a été prise en compte. Les résultats et les coûts ont été évalués en présumant l’administration du PNEU-P-23 seul, et autrement, l’administration du PNEU-C-13 → PNEU-P-23 : 1) à tous les adultes de ≥65 ans (*n* = 5,4 M); et 2) aux adultes immunodéprimés et à risque élevé de ≥65 ans (*n* = 3 M).

**Résultats:**

Pour la population 1, le PNEU-C-13 → PNEU-P-23 réduit de 1100 les cas de PI, de 7000 les cas de POC et de 135,8 M$ les coûts de ces maladies; les coûts de vaccination augmentent de 254,3 M$, et le coût par année de vie pondérée par la qualité (AVPQ) gagnée est de 35,484 $. Pour la population 2, le PNEU-C-13 → PNEU-P-23 réduit de 900 les cas de PI, de 6000 les cas de POC et de 120,3 M$ les coûts de ces maladies; les coûts de vaccination augmentent de 149,8 M$, et le coût par AVPQ gagnée est de 10,728 $.

**Conclusion:**

Élargir l’administration du PNEU-C-13 → PNEU-P-23 en finançant l’administration du PNEU-C-13 chez les Canadiens de ≥65 ans serait faire une utilisation efficace des ressources de soins de santé par rapport aux coûts.

## Introduction

*Streptococcus pneumoniae* (pneumococcus) is the leading cause of bacteremia, meningitis, and bacterial pneumonia in children and adults. Invasive pneumococcal disease (IPD)—including bacteremia and meningitis—is most common in the very young, the elderly, and specific risk groups, such as immunocompetent persons with chronic diseases (high risk) and those with immunocompromising conditions. Pneumococcal community-acquired pneumonia (CAP) can be both invasive and non-invasive; non-invasive/non-bacteremic pneumococcal pneumonia (NBPP) is more common but is difficult to diagnose.

Recent advances in diagnostic methods have been used to demonstrate the significant burden of NBPP in Canada (McNeil et al. 2014a, b), and to demonstrate the efficacy of the 13-valent pneumococcal conjugate vaccine (PCV13) against NBPP in the Community-Acquired Pneumonia Immunization Trial in Adults (CAPiTA) (Bonten et al. 2014). CAPiTA was a double-blind, randomized, placebo-controlled vaccine efficacy trial that enrolled approximately 85,000 immunocompetent subjects aged ≥ 65 years in the Netherlands. The primary objective of CAPiTA was to evaluate the efficacy of PCV13 against first episode of vaccine-type pneumococcal CAP; secondary objectives were to evaluate the efficacy of PCV13 against the first episode of vaccine-type NBPP and the first episode of vaccine-type IPD.

To reduce the burden of pneumococcal disease in Canada, the National Advisory Committee on Immunization (NACI) recommends use of PCV13 and the 23-valent pneumococcal polysaccharide vaccine (PPV23) in a sequential schedule (PCV13 → PPV23) among adults aged ≥ 65 years and adults aged ≥ 18 years who are immunocompromised (National Advisory Committee on Immunization 2013, 2016). Provincial and Territorial immunization programs generally follow NACI recommendations, but require provincial vaccine advisory committee recommendations before Ministry of Health approval of funding and implementation (Alberta Health 1995-2014; Communicable Disease Control. Manitoba’s Immunization Program 2014; Department of Health New Brunswick 2014; Public Health Agency of Canada 2008, 2014a, b; Sahni et al. 2012; Government of Nunavut 2010; Government of Saskatchewan Ministry of Health 2014; Ministère de la Santé et des Services sociaux 2014; Nova Scotia Department of Health and Wellness 2013, 2014; Newfoundland and Labrador Department of Health 2014; Northwest Territories Health and Social Services 2009, 2014; Ontario Health Government 2011; Ontario Ministry of Health and Long-Term Care 2012; Public Health Institute of Quebec 2014; Prince Edward Island Department of Health and Wellness 2014; Yukon Health and Social Services 2012). Most Canadian provinces and territories fund PPV23 for the prevention of pneumococcal disease in adults aged ≥ 65 years, immunocompetent adults with ≥ 1 risk factor, and adults with immunocompromising medical conditions. Some Canadian provinces and territories also fund sequential use of PCV13 and PPV23 for adults aged ≥ 18 years with immunocompromising medical conditions.

The economic implications of funding the sequential vaccination schedule (i.e., PCV13 → PPV23) in a broader population of Canadian adults are, however, currently unknown. While the findings of several recent evaluations indicate that adult use of PCV13 (i.e., either use of PCV13 alone or sequential use prior to PPV23) has a reasonable cost-effectiveness profile, these studies were based on epidemiologic and economic inputs from other countries and thus may not be reflective of the Canadian experience (Van Hoek and Miller 2016; Blommaert et al. 2016; Stoecker et al. 2016; Rodriguez Gonzalez-Moro et al. 2016; De Wals et al. 2016; Hoshi et al. 2015; Mangen et al. 2015). Therefore, in consideration of new efficacy data from CAPiTA, existing NACI and provincial vaccine advisory committee recommendations, and current funding for PCV13 and PPV23, an evaluation was undertaken to assess the cost-effectiveness of sequential use of PCV13 and PPV23—compared with PPV23 alone—in Canadian adults. Two alternative segments of the population were considered: (1) all adults aged ≥ 65 years at model entry and (2) immunocompromised and high-risk adults aged ≥ 65 years at model entry.

## Methods

### Model description

The model utilizes a microsimulation framework and a Markov-type process to depict expected lifetime risks, consequences, and costs of IPD and all-cause pneumonia, as well as the expected impact of vaccination, in the targeted populations of Canadian adults (Online Supplement—Model Schematic). Upon model entry, each person’s age (in one-year increments), risk profile (i.e., low risk [immunocompetent without chronic comorbidities], high risk [immunocompetent with ≥ 1 chronic comorbidity], or immunocompromised), and history of vaccination with PPV23 are assigned. Persons may transition to a higher risk group during the modeling horizon based on age-specific probabilities of developing new chronic medical conditions (low to high) or new immunocompromising conditions (low to immunocompromised, high to immunocompromised). Persons may receive PPV23, PCV13 → PPV23, or neither vaccine strategy at model entry; vaccine coverage may vary by age, risk profile, and vaccination history.

Expected clinical outcomes and economic costs are estimated for each person on an annual basis, based on age, risk profile, vaccination status, vaccination strategy (i.e., PPV23 or PCV13 → PPV23), and time since vaccination. IPD is stratified by condition (bacteremia vs. meningitis), and all-cause pneumonia is stratified by setting of care (inpatient vs. outpatient). Persons vaccinated at model entry or subsequently—or prior to model entry—may be at lower risk of future IPD and all-cause pneumonia. The magnitude of vaccine-associated risk reduction depends on clinical presentation (i.e., IPD or all-cause pneumonia), as well as the vaccine(s) administered, time since vaccination, age, and risk profile. Risk of death from IPD, all-cause pneumonia requiring inpatient care, and other causes (i.e., other than IPD and all-cause pneumonia) depends upon age and risk profile.

Expected costs of medical treatment for IPD and all-cause pneumonia are generated based on unit costs in relation to the setting of care (i.e., inpatient vs. outpatient), age, and risk profile. Costs of vaccination—including vaccine cost and administration—are tallied at model entry and at the time of subsequent vaccinations, as appropriate. Clinical outcomes and economic costs are projected over remaining years of life for each person in the model population for the vaccination strategies considered, and include numbers of cases of IPD (bacteremia and meningitis) and all-cause pneumonia (inpatient and outpatient), deaths due to IPD and all-cause pneumonia, life-years (unadjusted and quality-adjusted), costs of medical treatment for IPD and all-cause pneumonia, and costs of vaccination.

### Model estimation

Rates of disease, vaccine effectiveness, case-fatality rates, utilities, and disease-specific costs were estimated for age-specific (18–49, 50–64, 65–74, 75–84 and 85–99 years) and risk-specific (low risk, high risk, immunocompromised) subgroups based on data from published and secondary sources, and from these point-estimates, techniques of linear interpolation and extrapolation were used to project values for all ages in one-year increments. Rates of all-cause pneumonia requiring inpatient care and invasive disease (i.e., bacteremia and meningitis) were derived from the Canadian Institute for Health Information (CIHI) (Chiltern/OXON 2011) and were adjusted (i.e., reduced) to account for expected herd effects from the PCV13 pediatric program. The latter were estimated based on observations from the PCV7 experience in Canada over the past decade; the percentage of all-cause pneumonia attributable to PCV13 serotypes was assumed to decline from 8% in year 1 of the modeling horizon to 2% in year 5 (and beyond).

Costs of inpatient care for bacteremia, meningitis, and all-cause pneumonia were based on data from the CIHI and the Ontario Case Costing Initiative Database (OCCI, Ontario Case Costing Initiative 2016). For all-cause pneumonia, the cost of hospitalization was assumed to be the same irrespective of the causative pathogen (i.e., for pneumococcal and non-pneumococcal pneumonia). While the assumed unit costs are higher than those for all-cause pneumonia reported elsewhere, they are largely consistent with estimates for pneumococcal pneumonia from other sources (OCCI, Ontario Case Costing Initiative 2016) (Ontario Case Costing Initiative Database), which was deemed to be appropriate given the focus of this evaluation (i.e., on the prevention of pneumococcal pneumonia). We also note that the assumed unit costs for pneumonia requiring inpatient care are comparable to those reported in a recent Canadian evaluation of hospitalizations for influenza, in which the authors concluded that the cost of such hospitalizations is higher than previously reported (Ng et al. 2017). Medical costs of all-cause pneumonia requiring outpatient care only were based on the study by Morrow et al., adjusted for inflation (Morrow et al. 2007). The price of PPV23 was set at $11.00 per dose, which was based on 2016 IMS data and an assumed discount to approximate the confidential government contract price. The price used for PCV13 was the Pfizer confidential contract price, which is markedly higher than that for PPV23. Vaccine administration cost ($15.59) was based on published data (Skowronski et al. 2006).

A detailed description of methods employed to estimate all model parameter values is set forth in the online supplement (Online Supplement—Methods of Model Estimation), along with a table listing all measures, parameters on which all such measures were based (in full or in part), and corresponding sources/assumptions (Online Supplement—Listing of Measures, Parameters, and Sources/Assumptions). A summary of key model parameter values is provided in Tables 1 and 2.

**Table 1:** Estimates of population size, disease rates, case-fatality rates, and associated costs*

	Age/risk profile															Sources
	18–49 years			50–64 years			65–74 years			75–84 years			≥ 85 years			
	Low	High	Immuno-comp.	Low	High	Immuno-comp.	Low	High	Immuno-comp.	Low	High	Immuno-comp.	Low	High	Immuno-comp.	
No. of Canadian adults in:	10.4	4.7	0.3	4.0	2.9	0.5	1.4	1.3	0.3	0.7	0.7	0.2	0.3	0.3	0.1	Statistics Canada (2013), NHIS (2012)
Annual disease incidence^b^ (per 100 K)																Demczuk et al. (2013), Rudnick et al. (2013), Kellner et al. (2009), Kyaw et al. (2005), McNeil et al. (2012), Chiltern/OXON Report on file
Bacteremia	0.9	7.0	47.5	2.7	13.4	58.8	3.8	14.4	54.0	4.4	13.5	38.9	4.6	12.7	28.7	
Meningitis	0.05	0.37	2.50	0.14	0.70	3.09	0.20	0.76	2.84	0.23	0.71	2.05	0.24	0.67	1.51	
ACP																McNeil et al. (2012, 2014a, b), Griffin et al.(2013), Marrie and Huang (2005), Kyaw (2005), Chiltern/OXON Report on file
Inpatient	47	326	1974	108	477	2121	252	885	3329	590	1842	5952	913	2729	8181	
Outpatient	45	323	2109	88	431	2032	152	568	2250	276	873	2842	308	898	2506	
Annual mortality/case-fatality (per 100)																
Bacteremia	6.9	8.7	10.4	10.6	13.3	15.9	13.6	17.0	20.4	17.6	22.0	26.4	46.4	57.9	69.5	McNeil et al. (2014a, b), Statistics Canada (2013), NHIS (2012), Smith et al. (2008), Lexau et al. (2005), Robinson et al. (2001)
Meningitis	6.9	8.7	10.4	10.6	13.3	15.9	13.6	17.0	20.4	18.1	22.6	27.1	50.3	62.9	75.4	
ACP requiring inpatient care	3.4	4.3	5.2	6.7	8.4	10.1	8.6	10.8	13.0	11.6	14.5	17.4	17.8	22.3	26.7	
Medical care costs (per case)																Chiltern/OXON Report on file
Requiring inpatient care																
Bacteremia	$44,747	$54,174	$55,061	$44,627	$50,514	$58,934	$33,023	$44,980	$46,954	$35,465	$41,857	$46,355	$33,487	$45,442	$42,628	
Meningitis	$25,604	$30,998	$31,505	$25,535	$28,904	$33,721	$22,960	$31,273	$32,645	$24,658	$29,101	$32,229	$23,282	$31,594	$29,638	
ACP^c^	$14,605	$16,556	$21,403	$14,098	$16,001	$21,845	$17,347	$19,750	$25,591	$16,555	$20,234	$24,665	$16,691	$20,537	$23,294	
Requiring outpatient care only																
ACP	$96	$98	$113	$95	$97	$112	$95	$96	$112	$94	$96	$111	$94	$96	$111	Morrow et al. (2007)
Vaccination (per person)																Proactive Pharma Solutions 2014^d^ Pfizer Canada Skowronski et al. (2006)
PPV23	$11.00	$11.00	$11.00	$11.00	$11.00	$11.00	$11.00	$11.00	$11.00	$11.00	$11.00	$11.00	$11.00	$11.00	$11.00	
PCV13	Confidential Contact price															
Administration	$15.59	$15.59	$15.59	$15.59	$15.59	$15.59	$15.59	$15.59	$15.59	$15.59	$15.59	$15.59	$15.59	$15.59	$15.59	

NHIS: National Health Interview Survey

^a^Estimates correspond to mid-point of age range

^b^Rates in year 5 of modeling horizon, fully adjusted for herd effects

^c^Unit cost was assumed to be the same for all cases of ACP, irrespective of the causative pathogen (see supplement material for more details)

^d^The price of PPV23 was set at $11.00 per dose, which was based on 2016 IMS data and an assumed discount to approximate the confidential government contract price

**Table 2:** Effectiveness of PCV13 and PPV23

	PCV13, by no. of years					PPV23, by no. of years					Sources
	Since receipt of vaccine^b^					Since receipt of vaccine^b^					
	1	5	10	15	20	1	5	10	15	20	
IPD (due to vaccine serotypes^a^)											
Age/risk profile											
18–49											
Low/high risk	85%	85%	66%	35%	0%	93%	68%	22%	3%	0%	
Immunocompromised	66%	66%	52%	27%	0%	21%	17%	7%	1%	0%	
50–64 years											
Low/high risk	82%	82%	62%	31%	0%	87%	60%	17%	2%	0%	PPSV23: Smith et al. (2008), Shapiro et al. (1991)
Immunocompromised	64%	64%	48%	24%	0%	14%	12%	5%	1%	0%	
65–74 years											
Low/high risk	77%	77%	51%	22%	0%	77%	44%	9%	1%	0%	PCV13: Bonten et al. (2014), Klugman et al. (2003)
Immunocompromised	60%	60%	40%	17%	0%	1%	1%	0%	0%	0%	
75–84 years											
Low/high risk	72%	72%	41%	9%	0%	68%	31%	3%	0%	0%	
Immunocompromised	56%	56%	32%	7%	0%	0%	0%	0%	0%	0%	
≥ 85 years											
Low/high risk	68%	68%	5%	0%	0%	59%	20%	0%	0%	0%	
Immunocompromised	53%	53%	4%	0%	0%	0%	0%	0%	0%	0%	
ACP											
Age/risk profile											
18–49 years											
Low/high risk	4%	4%	3%	1%	0%	0%	0%	0%	0%	0%	
Immunocompromised	3%	3%	2%	1%	0%	0%	0%	0%	0%	0%	
50–64 years											
Low/high risk	4%	4%	3%	1%	0%	0%	0%	0%	0%	0%	
Immunocompromised	3%	3%	2%	1%	0%	0%	0%	0%	0%	0%	
65–74 years											
Low/high risk	4%	4%	2%	1%	0%	0%	0%	0%	0%	0%	PPSV23: Cho et al. (2013), Smith et al. (2013), Fry et al. (2002)
Immunocompromised	2%	2%	2%	1%	0%	0%	0%	0%	0%	0%	
75–84 years											
Low/high risk	3%	3%	2%	1%	0%	0%	0%	0%	0%	0%	PCV13: Bonten et al. (2014), McNeil et al. (2014a, b), Klugman (2003)
Immunocompromised	2%	2%	1%	0%	0%	0%	0%	0%	0%	0%	
≥ 85 years											
Low/high risk	3%	3%	1%	0%	0%	0%	0%	0%	0%	0%	
Immunocompromised	2%	2%	1%	0%	0%	0%	0%	0%	0%	0%	

^a^Serotype coverage (year 1): 68% for PPV23, 38% for PCV13

^b^Based on vaccine waning only; does not reflect changing serotype coverage over time

### Analyses

Base-case: Clinical outcomes and economic costs were projected over the lifetime for two alternative populations: all persons aged ≥ 65 years at model entry (*n* = 5.4M), and immunocompromised and high-risk persons aged ≥ 65 years (*n* = 3.0M) at model entry. For each of these populations, outcomes and costs were evaluated under two vaccination strategies: use of PPV23 and, alternatively, use of PCV13 → PPV23 among persons in the populations of interest. Vaccination rates were assumed to vary by age and risk, as follows: age 65–74 years, low risk—44%; age 75–99 years, low risk—65%; age 65–74 years, high-risk/immunocompromised—60%; age 75–99 years, high-risk/immunocompromised—65% (Public Health Institute of Quebec 2014).

Clinical outcomes and economic costs were simulated a total of 500 times, and each simulation included a population of 7.5 million persons (an approximate minimum number of persons required to produce stable results for all model outcomes in each age and risk group for each given vaccination strategy). Model populations were standardized to reflect the age, risk profile, and size of the Canadian populations of interest. Analyses were conducted from the perspective of the Canadian healthcare system; accordingly, only direct costs associated with the provision of medical care for IPD and all-cause pneumonia, and the costs of vaccination, were considered. Costs (2014 CAN$) and life-years were discounted at a 5% annual rate (Canadian Agency for Drugs and Technologies in Health 2006).

Sensitivity: One-way deterministic sensitivity analyses were undertaken to evaluate the potential impact of parameter value uncertainty on study results. In these analyses, key model parameter values were varied, each in turn, as follows: percentage of PCV13-type all-cause pneumonia in year 1 of modeling horizon assumed to be 5% and, alternatively, 10%; cost of hospitalization for all-cause pneumonia assumed to be 50% of base-case values; VE-PCV13 against NBPP in years 1–5 of modeling horizon, assumed to be 14% and, alternatively, 65% based on corresponding 95% confidence interval from CAPiTA (Bonten et al. 2014); durability of PCV13, assumed to persist at initial level for 8 years (assumption); herd effect on adult IPD rate assumed to be lower by 50% in each year of the modeling horizon (assumption); disutilities associated with IPD and all-cause pneumonia, based on values used in economic evaluations by Smith et al. (2012, 2013). Probabilistic sensitivity analysis (*n* = 500 replications) was employed to account for uncertainty surrounding disease rates and costs, case-fatality rates, and vaccine effectiveness and other key model parameters in estimation of clinical outcomes, economic costs, and incremental cost-effectiveness ratios.

## Results

### All persons aged ≥ 65 years

Base-case analysis: With use of PPV23 alone, the expected lifetime numbers of cases of disease among all persons aged ≥ 65 years at model entry (*n* = 5.4 million) totaled: IPD, 10,000; all-cause pneumonia requiring inpatient care, 1,735,000; and all-cause pneumonia requiring outpatient care only, 707,000 (Table 3). Expected lifetime medical care costs totaled $25.0 billion and vaccination costs $82.9 million. With use of PCV13 → PPV23, the expected lifetime numbers of cases of disease in this same population totaled: IPD, 8900; all-cause pneumonia requiring inpatient care, 1,730,000; and all-cause pneumonia requiring outpatient care only, 705,000. Expected lifetime medical care costs totaled $24.9 billion, and vaccination costs, $337.2 million.

**Table 3:** Cost-effectiveness of PCV13 → PPV23 vs. PPV23 in Canadian adults

	Use of PPV23 alone			Use of PCV13 → PPV23			Difference		
		95% CI			95% CI			95% CI	
	Mean	LCL	UCL	Mean	LCL	UCL	Mean	LCL	UCL
A. All persons aged ≥ 65 years at model entry (*N* = 5.4M)									
No. of cases									
IPD (in thousands)	9.951	9.312	10.194	8.871	8.310	9.098	− 1.080	− 1.565	− 0.513
ACP (in millions)									
Requiring inpatient care	1.735	1.715	1.743	1.730	1.713	1.741	− 0.005	− 0.006	0.003
Requiring outpatient care	0.707	0.660	0.719	0.705	0.658	0.716	− 0.002	− 0.003	0.001
No. of deaths (in millions)	0.363	0.355	0.403	0.362	0.355	0.401	− 0.001	− 0.002	0.000
Total costs (in billions)									
Medical care	25.000	24.382	25.472	24.865	24.313	25.315	− 0.136	− 0.138	− 0.010
Vaccination	0.083	0.083	0.083	0.337	0.337	0.337	0.254	0.254	0.255
Medical + vaccination	25.083	24.482	25.560	25.202	24.655	25.687	0.119	0.101	0.241
Life-years (discounted, per person)	7.8892	7.8652	7.8941	7.8903	7.8684	7.8942	0.0011	− 0.0025	0.0047
Quality-adjusted life-years (discounted, per person)	5.0782	5.0656	5.0802	5.0788	5.0667	5.0810	0.0006	− 0.0016	0.0023
Healthcare system perspective									
Cost per life-year gained	–	–	–	–	–	–	$20,227	–	–
Cost per quality-adjusted life-year gained	–	–	–	–	–	–	$35,484	–	–
B. Immunocompromised and high-risk persons aged ≥ 65 years at model entry (*N* = 3.0M)									
No. of cases									
IPD (in thousands)	7.373	7.064	7.651	6.473	6.172	6.680	− 0.901	− 1.240	− 0.625
ACP (in millions)									
Requiring inpatient care	1.209	1.202	1.216	1.205	1.197	1.210	− 0.004	− 0.006	− 0.003
Requiring outpatient care	0.503	0.490	0.517	0.501	0.486	0.514	− 0.002	− 0.005	− 0.002
No. of deaths (in millions)	0.248	0.234	0.262	0.247	0.233	0.262	− 0.001	− 0.002	− 0.001
Total costs (in billions)									
Medical care	18.574	18.205	18.937	18.453	18.061	18.838	− 0.120	− 0.153	− 0.097
Vaccination	0.049	0.049	0.049	0.199	0.199	0.199	0.150	0.150	0.150
Medical + vaccination	18.623	18.231	19.010	18.652	18.273	19.011	0.029	− 0.006	0.053
Life-years (discounted, per person)	7.2983	7.2825	7.3146	7.2999	7.2824	7.3155	0.0016	− 0.0004	0.0041
Quality-adjusted life-years (discounted, per person)	4.6480	4.6388	4.6564	4.6489	4.6399	4.6574	0.0009	− 0.0086	0.0104
Healthcare system perspective									
Cost per life-year gained	–			–			$6056		
Cost per quality-adjusted life-year gained	–			–			$10,728		

Accordingly, the use of PCV13 → PPV23 would reduce: expected lifetime cases of IPD by 1100; expected lifetime cases of pneumonia requiring inpatient care by 4800; expected lifetime cases of pneumonia requiring outpatient care by 2200; expected total disease-related deaths by 1000; and expected lifetime total medical care costs by $135.6 million. Total vaccination costs, however, would increase by $254.3 million, and thus total overall costs would be higher by $118.7 million. Cost per QALY gained (healthcare system perspective) would be $35,484.

**Fig. 1:**
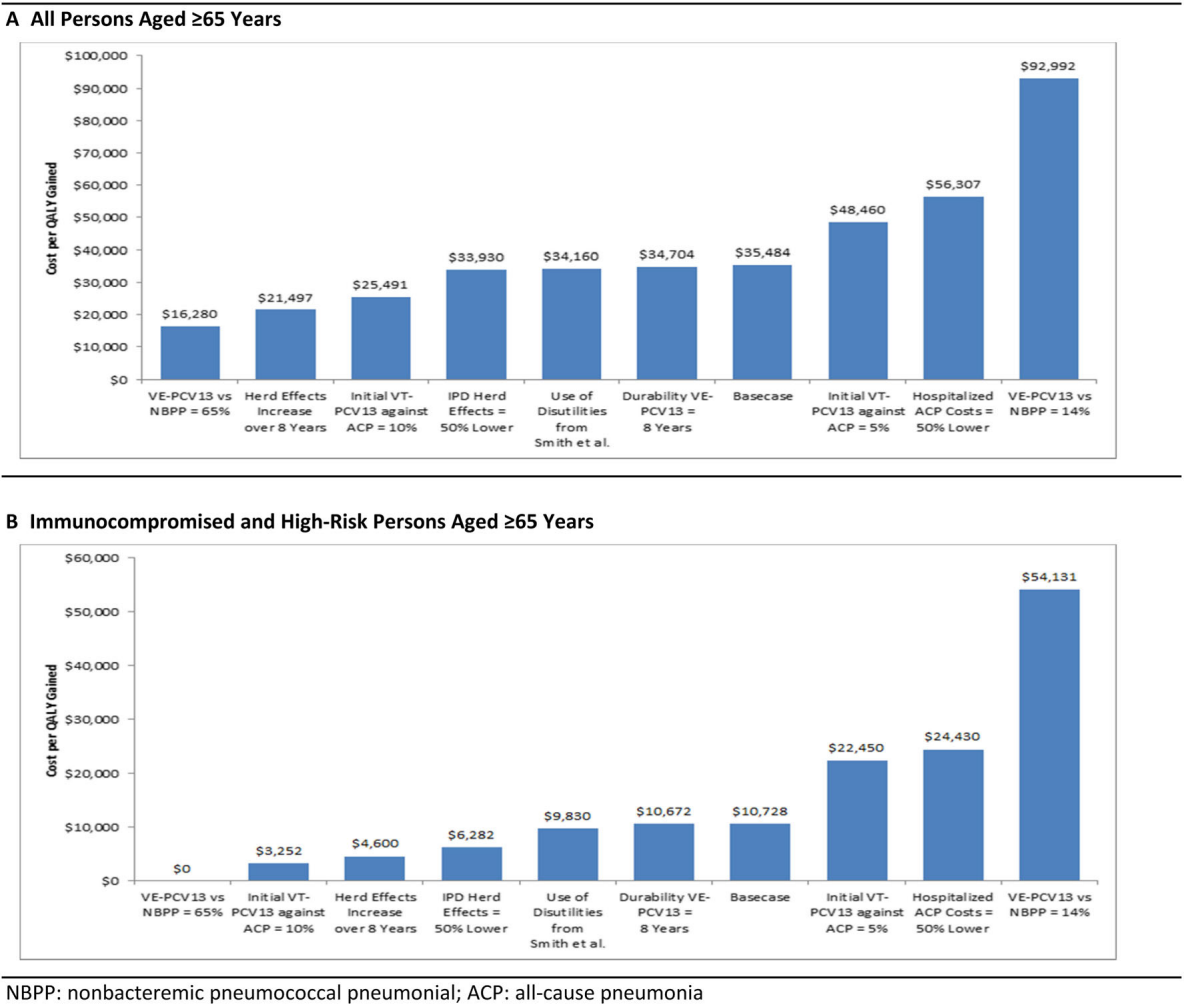
One-way deterministic sensitivity analyses for cost per QALY gained with PCV13 → PPV23 vs PPV23 alone

**Fig. 2:**
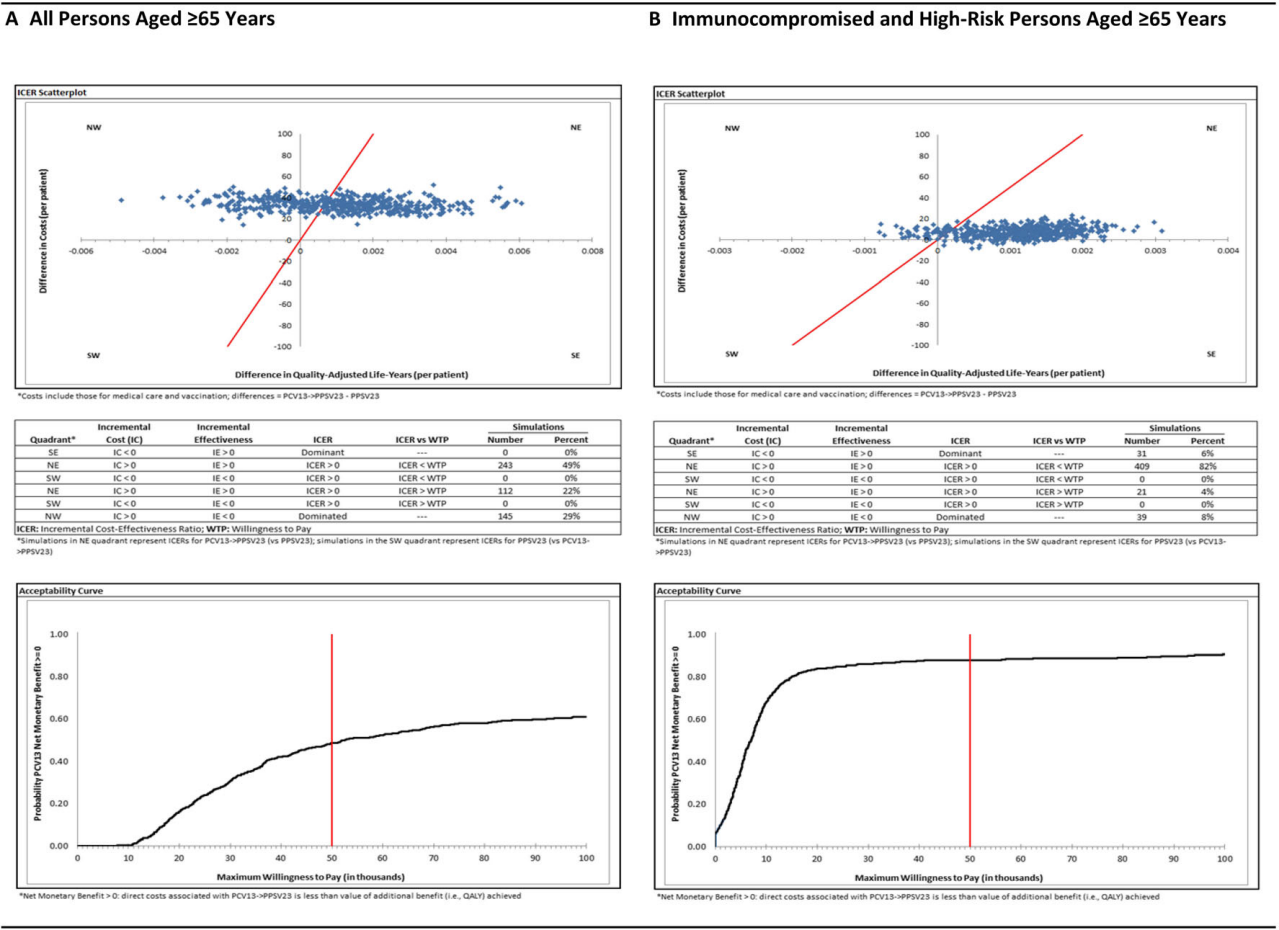
Scatterplots and acceptability curves for cost per QALY gained with PCV13 → PPV23 vs PPV23 alone

Sensitivity analyses: In all one-way deterministic sensitivity analyses, with two exceptions, cost per QALY gained with PCV13 → PPV23 (vs. PPV23 alone) was less than the presumed maximum willingness to pay (WTP $50,000); when assuming the cost of hospitalization for all-cause pneumonia was lower by 50%, cost per QALY gained was $56,307, and when assuming effectiveness of PCV13 against vaccine-type NBPP was 14% (instead of 45%, based on the CAPiTA study), cost per QALY gained was $92,992 (Fig. 1). In probabilistic sensitivity analyses, 49% of the 500 simulations generated cost-effectiveness ratios for PCV13 → PPV23 (vs. PPV23 alone) that were located in the northeast quadrant of the scatter plot and were less than the maximum WTP; an additional 22% of the simulations generated ratios that were located in the northeast quadrant but were higher than the maximum WTP (Fig. 2).

### Immunocompromised and high-risk persons aged ≥ 65 years

Base-case analysis: With use of PPV23 alone, the expected lifetime numbers of cases of disease among immunocompromised and high-risk persons aged ≥ 65 years at model entry (*n* = 3.0 million) totaled as follows: IPD, 7400; all-cause pneumonia requiring inpatient care, 1,209,000; and all-cause pneumonia requiring outpatient care only, 503,000. Expected lifetime medical care costs totaled $18.6 billion, and vaccination costs, $48.8 million. With use of PCV13 → PPV23, the expected lifetime numbers of cases of disease in this same population totaled as follows: IPD, 6500; all-cause pneumonia requiring inpatient care, 1,205,000; and all-cause pneumonia requiring outpatient care only, 501,000. Expected lifetime medical care costs totaled $18.5 billion, and vaccination costs, $198.6 million.

Accordingly, the use of PCV13 → PPV23 would reduce: expected lifetime cases of IPD by 900; expected lifetime cases of pneumonia requiring inpatient care by 4100; expected lifetime cases of pneumonia requiring outpatient care by 1900; expected total disease-related deaths by 1000; and expected total lifetime medical care costs by $120.3 million. Total vaccination costs, however, would increase by $149.8 million, and thus total overall costs would be higher by $29.5 million. Cost per QALY gained (healthcare system perspective) would be $10,728.

Sensitivity analyses: In all one-way deterministic sensitivity analyses, with one exception, PCV13 → PPV23 was either dominant versus PPV23 alone or was associated with a cost per QALY gained that was less than the maximum WTP; when assuming effectiveness of PCV13 against vaccine-type NBPP was 14% (instead of 45%, based on the CAPiTA study), cost per QALY gained was $54,131. In probabilistic sensitivity analyses, 82% of the 500 simulations generated cost-effectiveness ratios for PCV13 → PPV23 (vs. PPV23 alone) that were located in the northeast quadrant of the scatter plot and were less than the maximum WTP; an additional 6% of simulations generated ratios that were located in the southeast quadrant of the scatterplot (i.e., lower costs and higher QALYs).

## Discussion

An evaluation was undertaken to assess the cost-effectiveness of PCV13 → PPV23—compared to PPV23 alone—in Canadian adults. Our findings indicate that implementation of an expanded vaccination program in both evaluated groups (i.e., all adults aged ≥ 65 years and immunocompromised/high-risk adults aged ≥ 65 years, respectively) would reduce the number of cases of pneumococcal disease and pneumococcal-related deaths and would be a cost-effective use of resources from the Canadian healthcare system perspective. These findings were projected assuming reasonable values for several key model parameters and were robust when varying key model parameters in deterministic and probabilistic sensitivity analyses.

We note that the cost-effectiveness of expanding vaccination to include high-risk adults aged ≥ 65 years is favourable relative to expanding vaccination to include high-risk and low-risk adults aged ≥ 65 years. For each dollar spent vaccinating high-risk (vs. low-risk) persons, the numbers of cases and deaths prevented, and the associated cost offsets, are higher and thus value is greater. We also note, however, that such benefits must be evaluated against the effectiveness of broad age-based recommendations (vs. age- and risk-based recommendations) in attaining higher vaccine coverage levels, and that when including the low-risk population, the incremental cost-effectiveness ratio remained acceptable by conventional standards. We also note that notwithstanding differences in model structure, model population, methods of model estimation, and vaccination strategies, our conclusions are largely consistent with those from recent evaluations in which adult PCV13 use was found to have a reasonable cost-effectiveness profile based on current disease epidemiology (Van Hoek and Miller 2016; Blommaert et al. 2016; Stoecker et al. 2016; Rodriguez Gonzalez-Moro et al. 2016; De Wals et al. 2016; Hoshi et al. 2015; Mangen et al. 2015).

A limitation of this assessment is the uncertainty surrounding some of the parameter estimates. The areas of greatest uncertainty are the incidence rate of vaccine-type pneumonia in Canada, the assumed effectiveness of vaccination with PPV23 against IPD and all-cause pneumonia in older adults, and the persistency of vaccine benefits. For this analysis, the proportion of all-cause pneumonia caused by vaccine types was 8.0% among adults aged ≥ 65 years (personal communication Dr. McNeil 2015). However, this proportion is dependent on diagnostic methods used and is likely conservative (personal communication Dr. McNeil, submitted for publication).

Available data on levels and duration of PPV23 effectiveness against IPD among immunocompetent persons, on an overall basis, as well as by age, risk, and time since receipt, are currently limited. We therefore utilized published figures from a cost-effectiveness study that incorporated expert opinion and data from a pivotal case-control study as well as assumptions from other modeling exercises (Smith et al. 2008; Shapiro et al. 1991). There is evidence to suggest that the duration of protection conferred by PPV23 against IPD may be much shorter than that assumed in our evaluation based on data from the UK Health Protection Agency (Andrews et al. 2012). For PPV23 effectiveness against IPD in immunocompromised persons, we assumed the vaccine conferred little benefit in persons aged 50–64 years and no benefit in persons aged ≥ 65 years, consistent with a meta-analysis of relevant trial data, data from other studies, and assumptions employed in other modeling exercises (Smith et al. 2008; French et al. 2000; World Health Organization 2008; Fry et al. 2002). For PPV23 effectiveness against all-cause pneumonia, we assumed the vaccine conferred no benefit, consistent with the findings of recent meta-analyses (Kraicer-Melamed et al. 2016a, b; Schiffner-Rohe et al. 2016).

For PCV13, estimates of effectiveness were based primarily on the CAPiTA trial (Bonten et al. 2014). Assumptions regarding the rate of change in PCV13 effectiveness by age and time since receipt were based on data for PPV23, and effectiveness in immunocompromised persons was estimated from a single trial of children with HIV and children without HIV in South Africa (Klugman et al. 2003). The duration of PCV13 efficacy beyond follow-up in CAPiTA is unknown. Because PCV13 elicits a T-cell-dependent immune response, we assumed the waning to be half that of PPV23 after the stable efficacy period extending through the end of the 5-year period following receipt (Smith et al. 2008; Shapiro et al. 1991; Andrews et al. 2012). Assumptions underlying PCV13 effectiveness were evaluated in one-way deterministic sensitivity analyses, which yielded results largely comparable to those from base-case analyses. Moreover, we note that CAPiTA was a clinical efficacy study and thus was not subject to the vagaries of clinical practice. Accordingly, vaccine efficacy findings from CAPiTA may not be fully reflective of vaccine effectiveness in clinical practice*.*

Another area of parameter uncertainty is the estimated incidence of IPD and all-cause pneumonia among adults of all ages, not only current levels but also future levels of disease that may be prevented with vaccination. Rates of disease—estimated using nationally representative sources—were adjusted for projected herd effects from widespread use of PCV13 in children. The extent to which herd effects will impact future rates of vaccine-type disease are, however, largely unknown at this time. We note that assumptions employed in base-case analyses were conservative, assuming levels of vaccine-preventable disease to be low at model entry and to further decline over the subsequent years of the modeling horizon, and that these assumptions were explored in one-way deterministic sensitivity analyses. In addition, this study included only the acute (short-term) costs, but pneumonia has been found to have a significant impact on long-term morbidity and mortality, particularly among older adults; the true cost of pneumonia to the Canadian healthcare system was most likely underestimated (Eurich et al. 2015; Dharmarajan et al. 2013; Sandvall et al. 2013; Wasser et al. 2013). We did not consider vaccine-related adverse events in our analyses as the safety profiles of PCV13 and PPV23 have been reported to be similar, and serious adverse events associated with PCV13 and PPV23 vaccination are uncommon (Jackson et al. 2013a, b; Khoie et al. 2011; Stoecker et al. 2016).

Finally, our model, like all simulation models, simplifies reality to some extent. Our model does not, for example, take into account serotype replacement over time. Also, while our model accounts for indirect epidemiologic effects of childhood PCV13 vaccination, our model does not account for indirect epidemiologic effects of adult PCV13 vaccination (due to uncertainty regarding the precise nature of the relationship between vaccine coverage and the magnitude of such indirect effects), which undoubtedly confers a conservative bias against the benefits of vaccination.

## Conclusion

Most Canadian provinces and territories fund PPV23 for the prevention of pneumococcal disease in adults aged ≥ 65 years, immunocompetent adults with ≥ 1 risk factor, and adults with immunocompromising medical conditions. Some Canadian provinces and territories also fund sequential use of PCV13 and PPV23 for adults with immunocompromising medical conditions. Despite these efforts, the clinical burden of pneumococcal disease among Canadian adults remains substantial, especially among older adults who are more vulnerable and have more complex comorbidity profiles that put them at higher risk of disease. The results of this research suggest that implementing a comprehensive immunization program with PCV13 followed by PPV23 (in lieu of PPV23 alone) for the prevention of pneumococcal disease among all Canadian adults aged ≥ 65 years (especially those who are immunocompromised or at high risk) would be a cost-effective use of healthcare resources.

## Electronic supplementary material

ESM 1 (DOC 352 kb)
