# Supplementary material for: Cost-effectiveness of alternative strategies for use of 13-valent pneumococcal conjugate vaccine (PCV13) in Canadian adults
Source: Can J Public Health. 2018 May 9;109(5-6):756–68. doi: 10.17269/s41997-018-0050-9 (PMC6267650; doi:10.17269/s41997-018-0050-9)
Supplement: Supplementary file 1 — (DOC 352 kb) [file 41997_2018_50_MOESM1_ESM.doc]

**Cost-Effectiveness of Alternative Strategies for Use of**

**13-Valent Pneumococcal Conjugate Vaccine (PCV13) in Canadian Adults:**

**Online Supplement -- Model Schematic**


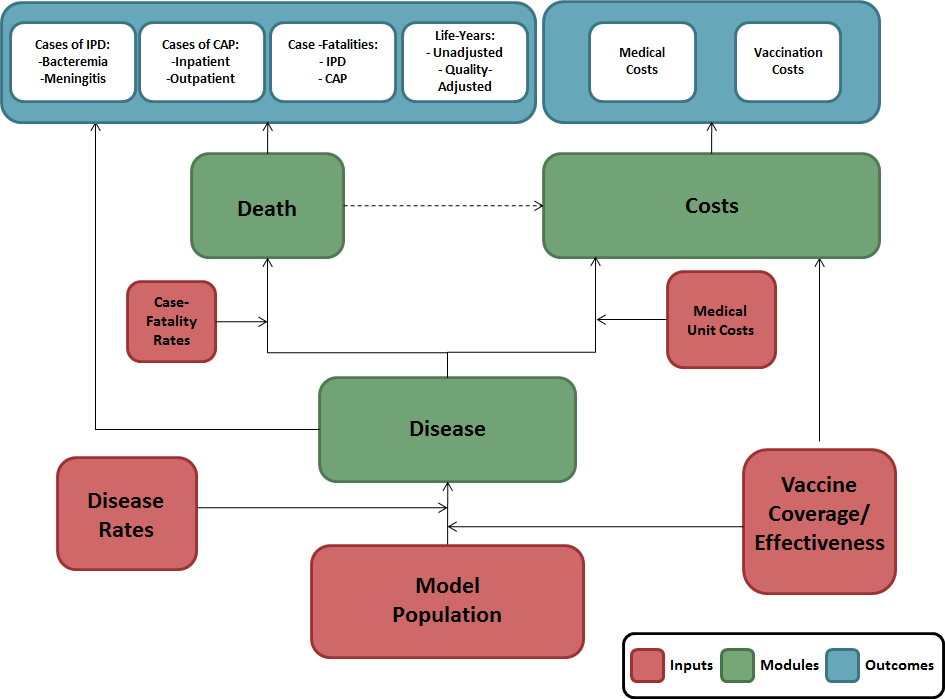


**Cost-Effectiveness of Alternative Strategies for Use of**

**13-Valent Pneumococcal Conjugate Vaccine (PCV13) in Canadian Adults:**

**Online Supplement -- Methods of Model Estimation**

**Population**

Estimates of the size of the Canadian population aged ≥65 years, in one-year increments, were obtained from Statistics Canada for calendar year 2013 (Statistics Canada 2013). The percentages of persons in each age group classified as low-risk, high-risk, or immunocompromised were estimated using data from the 2012 National Health Interview Survey (CDC NHIS 2012). This source was used because it provided detailed data on risk profiles by age, and Canadian data were not available for all age/risk groups; percentages for the 18-49 and 50-64 age and risk groups were validated against Canadian data.

Annual probabilities of transitioning from one risk group to another (i.e., from low to high, low to immunocompromised, high to immunocompromised) were estimated based on age-specific differences in the percentage of persons in each risk group. History of prior vaccination with PPV23 (i.e., at model entry), by age and risk profile, was based on published data (PHAC 2012, Quebec Public Health 2011, McNeil 2012); mean time since receipt of PPV23 was calculated using data from the 2012 NHIS (CDC NHIS 2012). For low-risk persons aged 18-64 years, history of prior vaccination with PPV23 was assumed to be zero.

**Rates of Disease**

**Invasive Pneumococcal Disease**

**Unadjusted for Herd Effects:** Annual age- and risk-specific rates of IPD (vaccine-type [VT] and non-vaccine-type [NVT])—assuming no pneumococcal vaccination among adults and no indirect “herd effects” (i.e., reductions in IPD) among adults due to widespread use of PCV13 in children—were based on 2012 passive surveillance data from the National Microbiology Laboratory - NML (Table 1) (Demczuk 2013). The data included cultures from all Canadian provincial and territorial public health and regional hospital laboratories in addition to surveillance data provided by the Laboratoire de santé publique du Québec, the Toronto Invasive Bacterial Diseases Network (TIBDN), which is an active surveillance, and the Provincial Laboratory for Public Health, Edmonton, Alberta (Demczuk 2013).

Overall rates were apportioned between persons in the low-risk, high-risk, and immunocompromised groups, based on corresponding relative-risk ratios from published data (Kyaw 2005). Bacteremia was assumed to account for 95% of all cases of IPD, with meningitis accounting for the remainder (Rudnick 2013, Kellner 2009). Because annual rates of disease are reported by age-groups spanning multiple years (e.g., 18-49 years, 50-64 years, 65-79 years, ≥80 years), piece-wise regression analysis was used to estimate a “smoothed” relationship between age-specific point estimates for each risk group, resulting in estimates of disease rates in one-year increments for low-risk, high-risk, and immunocompromised persons, respectively.

**Suppl Table 1: Rates of IPD (per 100,000), unadjusted for herd effects**

|  | **Bacteremia** | | | **Meningitis** | | |
| --- | --- | --- | --- | --- | --- | --- |
|  | **Risk Profile** | | | **Risk Profile** | | |
| **Age Group** | **Low** | **High** | **Immuno-**  **Compromised** | **Low** | **High** | **Immuno-**  **Compromised** |
| **18 – 49** | **1.4** | **10.8** | **33.8** | **0.07** | **0.57** | **1.78** |
| **50 – 64** | **2.9** | **22.8** | **71.1** | **0.15** | **1.20** | **3.74** |
| **65 – 74** | **2.7** | **21.2** | **66.1** | **0.14** | **1.12** | **3.48** |
| **75 – 84** | **3.4** | **26.4** | **82.3** | **0.18** | **1.39** | **4.33** |
| **85 – 99** | **4.2** | **32.6** | **101.9** | **0.22** | **1.72** | **5.36** |

**Adjusted for Herd Effects.** Baseline rates of IPD, as described above, were adjusted (i.e., reduced) to account for expected herd effects from widespread use of PCV13 in young children (Table 2). Herd effects were estimated on an age-specific basis by calculating the reductions in the rate of pneumococcal pneumonia observed in CIHI health records data during five years after the introduction of PCV7 in Canada (McNeil 2012, Chiltern/OXON Report on file). It was noted that the relative decline of 39% in pneumococcal pneumonia among persons aged ≥65 years from the CIHI health records database (McNeil 2012) was similar to the relative decline in IPD of ~40% that was observed in Calgary after the introduction of PCV7 between 2002 and 2009 (Ricketson 2014). Maximum values for herd effects were assumed to be obtained by year 5 of the modeling horizon and to persist at these levels thereafter.

**Suppl Table 2. Rates of IPD per 100,000 in year 5 of modeling horizon, adjusted for full herd effects**

| **Age** | **Bacteremia** | | | **Meningitis** | | |
| --- | --- | --- | --- | --- | --- | --- |
|  | **Risk Profile** | | | **Risk Profile** | | |
|  | **Low** | **High** | **Immuno-**  **Compromised** | **Low** | **High** | **Immuno-**  **Compromised** |
| **18 – 49** | **1.1** | **8.3** | **25.8** | **0.06** | **0.43** | **1.36** |
| **50 – 64** | **1.8** | **14.1** | **44.1** | **0.09** | **0.74** | **2.32** |
| **65 – 74** | **1.9** | **14.6** | **45.5** | **0.10** | **0.77** | **2.39** |
| **75 – 84** | **2.1** | **16.1** | **50.3** | **0.11** | **0.85** | **2.65** |
| **85 – 99** | **2.3** | **17.9** | **55.9** | **0.12** | **0.94** | **2.94** |

**All-Cause Pneumonia**

**Unadjusted for Herd Effects.** Age-specific annual rates of all-cause pneumonia requiring inpatient care—assuming no indirect “herd effects” among adults due to use of PCV13 in children—were based on data from the CIHI health records database for the 2009-2010 season (Table 3) (McNeil 2012, Chiltern/OXON Report on file). Age-specific annual rates of all-cause pneumonia requiring outpatient care only were derived by multiplying all-cause pneumonia inpatient rates (Chiltern/OXON Report on file) and the ratio of outpatient to inpatient rates abstracted from an Edmonton study (Marrie 2005). Age-specific rates of all-cause pneumonia were apportioned across risk groups and smoothed using the technique described above (Kyaw 2005).

**Suppl** Table 3. Rates of all-cause pneumonia (per 100,000), unadjusted for herd effects

|  | **Inpatient Pneumonia** | | | **Outpatient Pneumonia** | | |
| --- | --- | --- | --- | --- | --- | --- |
|  | **Risk Profile** | | | **Risk Profile** | | |
| **Age Group** | **Low** | **High** | **Immuno-**  **Compromised** | **Low** | **High** | **Immuno-**  **Compromised** |
| **18 – 49** | **26** | **206** | **642** | **55** | **434** | **1,354** |
| **50 – 64** | **55** | **434** | **1,355** | **67** | **526** | **1,640** |
| **65 – 74** | **126** | **986** | **3,076** | **82** | **644** | **2,010** |
| **75 – 84** | **280** | **2,196** | **6,852** | **136** | **1,068** | **3,331** |
| **85 – 99** | **807** | **6,341** | **19,788** | **257** | **2,018** | **6,296** |

**Adjusted for Herd Effects.** Baseline rates of all-cause pneumonia, as described above, were adjusted (i.e., reduced) to account for expected herd effects from widespread use of PCV13 in young children, which were based on CIHI health records during the period following the introduction of PCV7 in Canada; for example, a 13% reduction in all-cause pneumonia was observed in persons aged ≥65 years between 2004 and 2010 (Figure, Table 4) (Chiltern/OXON Report on File). In year 1 of the modeling horizon, the percentage of all-cause pneumonia due to PCV13 serotypes was estimated to be 8%, and that due to PCV7 serotypes, 1% (McNeil 2014); thus, 7% of all-cause pneumonia was assumed to be attributable to the six new serotypes in PCV13. The percentage of disease caused by the six new serotypes was assumed to decrease linearly (Griffin 2013) from 7% in year 1 to 1% in year 5, and to persist at 1% through the end of the modeling horizon. Disease caused by PCV7 serotypes was assumed to persist at 1% throughout the modeling horizon (McNeil 2014 personal communication).

**Suppl Figure 1. Percentage of all-cause pneumonia due to vaccine-type and non-vaccine-type serotypes during first 5 years of modeling horizon***


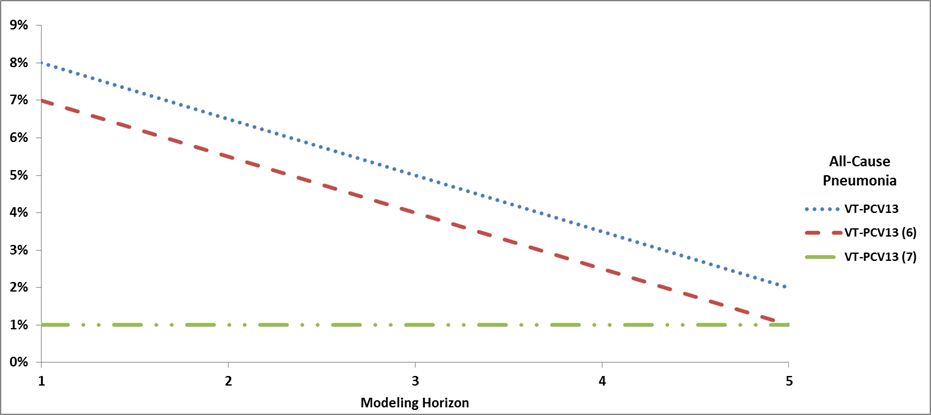


All-cause pneumonia (VT-6): 6 serotypes in PCV13 but not PCV7; all-cause pneumonia (VT-7): 7 serotypes in PCV13/PCV7

*From year 5 to end of modeling horizon, % of all-cause pneumonia caused by VT-6 was assumed to be constant at 1%

**Suppl** Table 4. Rates of all-cause pneumonia per 100,000 in year 5 of modeling horizon, adjusted for herd effects

| **Age** | **Inpatient Pneumonia** | | | **Outpatient Pneumonia** | | |
| --- | --- | --- | --- | --- | --- | --- |
|  | **Risk Profile** | | | **Risk Profile** | | |
|  | **Low** | **High** | **Immuno-**  **Compromised** | **Low** | **High** | **Immuno-**  **Compromised** |
| **18 – 49** | **20** | **158** | **493** | **41** | **319** | **997** |
| **50 – 64** | **47** | **370** | **1,154** | **47** | **372** | **1,162** |
| **65 – 74** | **99** | **774** | **2,416** | **60** | **468** | **1,461** |
| **75 – 84** | **227** | **1,782** | **5,562** | **93** | **730** | **2,277** |
| **85 – 99** | **471** | **3,700** | **11,547** | **146** | **1,150** | **3,589** |

**Case-Fatality Rates**

Age-specific case-fatality rates associated with IPD were based on a study of the clinical and economic burden of hospitalization due to *S. pneumococcus* septicemia and meningitis (McNeil 2014) (Table 5, Table 6). These age-specific fatality rates were adjusted for the risk groups based on published relative risks of death among high-risk and immunocompromised persons (Smith 2008, Lexau 2005, Robinson 2001) and corresponding population weights (Statistics Canada 2013, CDC NHIS 2012). Case-fatality associated with all-cause pneumonia requiring inpatient care was based on 30-day mortality rates among persons hospitalized for community-acquired pneumonia in Canada (Dr. McNeil, personal communication), and age-specific rates were allocated across risk groups based on published relative risks of death for the high-risk and immunocompromised groups (Smith 2008, Lexau 2005, Robinson 2001) and corresponding population weights (Statistics Canada 2013, CDC NHIS 2012).

For persons projected to develop pneumonia requiring outpatient care only, and for those projected to be free of IPD and pneumonia in a given year, the risk of death was calculated based on age- and risk-specific mortality rates from the general population, adjusted downward to account for potential death due to IPD and inpatient pneumonia (based on corresponding estimates of disease incidence and case-fatality rates) (Statistics Canada 2011). Regression analysis was used to smooth the relationship by age in each risk subgroup using the above-noted technique.

**Suppl Table 5.** Case-fatality rates for IPD (per 100)

|  | **Bacteremia** | | | **Meningitis** | | |
| --- | --- | --- | --- | --- | --- | --- |
|  | **Risk Profile** | | | **Risk Profile** | | |
| **Age Group** | **Low** | **High** | **Immuno-**  **Compromised** | **Low** | **High** | **Immuno-**  **Compromised** |
| **18 – 49** | **6.9** | **8.7** | **10.4** | **6.9** | **8.7** | **10.4** |
| **50 – 64** | **10.6** | **13.3** | **15.9** | **10.6** | **13.3** | **15.9** |
| **65 – 74** | **13.6** | **17.0** | **20.4** | **13.6** | **17.0** | **20.4** |
| **75 – 84** | **17.6** | **22.0** | **26.4** | **18.1** | **22.6** | **27.1** |
| **85 – 99** | **46.4** | **57.9** | **69.5** | **50.3** | **62.9** | **75.4** |

**Suppl** Table 6. Case-fatality rates for all-cause pneumonia (per 100)

|  | **Cases Requiring Inpatient Care** | | | **Cases Requiring Outpatient Care Only** | | |
| --- | --- | --- | --- | --- | --- | --- |
|  | **Risk Profile** | | | **Risk Profile** | | |
| **Age Group** | **Low** | **High** | **Immuno-**  **Compromised** | **Low** | **High** | **Immuno-**  **Compromised** |
| **18 – 49** | **3.4** | **4.3** | **5.2** | **---** | **---** | **---** |
| **50 – 64** | **6.7** | **8.4** | **10.1** | **---** | **---** | **---** |
| **65 – 74** | **8.6** | **10.8** | **13.0** | **---** | **---** | **---** |
| **75 – 84** | **11.6** | **14.5** | **17.4** | **---** | **---** | **---** |
| **85 – 99** | **17.8** | **22.3** | **26.7** | **---** | **---** | **---** |

**Vaccine Effectiveness**

**Vaccine-Type Invasive Pneumococcal Disease**

**PPV23.** Effectiveness of vaccination with PPV23 against VT-IPD—by age and time since vaccination—among low- and high-risk adults aged ≥50 years, and immunocompromised adults aged ≥65 years, was based on published data from Smith et al. (2008) (Table 7, Table 8):

- - Estimates reported by Smith and colleagues were based on a Delphi panel of experts, which relied primarily on data from Shapiro et al. (1991)
  - Estimates reported in Smith et al. (2008) are generally consistent with findings set forth in a technical report by the UK Health Protection Agency (Gungabissoon 2005)

Effectiveness of PPV23 in the first year following receipt and subsequently among immunocompetent adults aged 18-49 years was assumed to be the same as that for persons aged 50 years. For immunocompromised adults aged 18-50 years, initial effectiveness (21%) was based on data from Shapiro et al. (1991); for those aged 51-64 years, effectiveness was estimated by interpolating between values for persons aged 50 years (21%) and persons aged 69 years (0%) (Fry 2002). The assumed rate of decline in protection by time since vaccination for immunocompromised persons aged 18-64 years was based on data from Smith et al. (2008). Effectiveness of PPV23 was assumed to be the same irrespective of prior vaccination experience with either PPV23 or PCV13 (i.e., potential vaccine hyporesponsiveness was not considered).

**PCV13.** Effectiveness of PCV13 against VT-IPD for immunocompetent persons was based on the estimated effectiveness of PCV13 against VT-IPD among subjects in the CAPiTA trial (Bonten 2014, Hak 2008). Estimated effectiveness (75.0%) against VT-IPD was “anchored” to persons aged 73 years (mean age of study subjects in CAPiTA ), and protection was assumed to remain constant over the initial 5 years of the modeling horizon, based on the observation that vaccine effectiveness remained stable during the follow-up period (mean, 3.97 years) in CAPiTA. Rate of change in PCV13 effectiveness with age (i.e., for persons younger and older than 73 years of age) was equal to 50% of PPV23 values, and the rate of decline in PCV13 effectiveness over time (i.e., after the initial 5-year period) was assumed equal to 50% of PPV23 values beginning with the first year following receipt. Results from the CAPiTA trial correspond to low- and high-risk persons in the model population.

For immunocompromised adults, vaccine effectiveness was assumed to be 78% of corresponding values for the immunocompetent groups. This assumption was based on a trial of pneumococcal vaccination in children with and without HIV (Klugman 2003). Effectiveness of PCV13 was assumed to be the same irrespective of previous vaccination with either PPV23 or PCV13. Effectiveness of each vaccine (i.e., PPV23 and PCV13) across VT-serotypes was assumed to be the same.

**Suppl** Table 7. Effectiveness against VT IPD in the first year following vaccination

|  | **PCV13** | | | **PPV23** | | |
| --- | --- | --- | --- | --- | --- | --- |
|  | **Risk Profile** | | | **Risk Profile** | | |
| **Age Group** | **Low** | **High** | **Immuno-**  **Compromised** | **Low** | **High** | **Immuno-**  **Compromised** |
| **18 – 49** | **85%** | **85%** | **66%** | **93%** | **93%** | **21%** |
| **50 – 64** | **82%** | **82%** | **64%** | **87%** | **87%** | **14%** |
| **65 – 74** | **77%** | **77%** | **60%** | **77%** | **77%** | **1%** |
| **75 – 84** | **72%** | **72%** | **56%** | **68%** | **68%** | **0%** |
| **85 – 99** | **68%** | **68%** | **53%** | **59%** | **59%** | **0%** |

**Suppl Table 8. Effectiveness against VT IPD over time**

| **Age/Risk** | **PCV13** | | | | | **PPV23** | | | | |
| --- | --- | --- | --- | --- | --- | --- | --- | --- | --- | --- |
|  | **Years Since Receipt of Vaccine** | | | | | **Years Since Receipt of Vaccine** | | | | |
|  | **1** | **5** | **10** | **15** | **20** | **1** | **5** | **10** | **15** | **20** |
| **18 – 49** |  |  |  |  |  |  |  |  |  |  |
| **Low** | **85%** | **85%** | **66%** | **35%** | **0%** | **93%** | **68%** | **22%** | **3%** | **0%** |
| **High** | **85%** | **85%** | **66%** | **35%** | **0%** | **93%** | **68%** | **22%** | **3%** | **0%** |
| **Immuno-Compromised** | **66%** | **66%** | **52%** | **27%** | **0%** | **21%** | **17%** | **7%** | **1%** | **0%** |
| **50 – 64** |  |  |  |  |  |  |  |  |  |  |
| **Low** | **82%** | **82%** | **62%** | **31%** | **0%** | **87%** | **60%** | **17%** | **2%** | **0%** |
| **High** | **82%** | **82%** | **62%** | **31%** | **0%** | **87%** | **60%** | **17%** | **2%** | **0%** |
| **Immuno-Compromised** | **64%** | **64%** | **48%** | **24%** | **0%** | **14%** | **12%** | **5%** | **1%** | **0%** |
| **65 – 74** |  |  |  |  |  |  |  |  |  |  |
| **Low** | **77%** | **77%** | **51%** | **22%** | **0%** | **77%** | **44%** | **9%** | **1%** | **0%** |
| **High** | **77%** | **77%** | **51%** | **22%** | **0%** | **77%** | **44%** | **9%** | **1%** | **0%** |
| **Immuno-Compromised** | **60%** | **60%** | **40%** | **17%** | **0%** | **1%** | **1%** | **0%** | **0%** | **0%** |
| **75 – 84** |  |  |  |  |  |  |  |  |  |  |
| **Low** | **72%** | **72%** | **41%** | **9%** | **0%** | **68%** | **31%** | **3%** | **0%** | **0%** |
| **High** | **72%** | **72%** | **41%** | **9%** | **0%** | **68%** | **31%** | **3%** | **0%** | **0%** |
| **Immuno-Compromised** | **56%** | **56%** | **32%** | **7%** | **0%** | **0%** | **0%** | **0%** | **0%** | **0%** |
| **85 – 99** |  |  |  |  |  |  |  |  |  |  |
| **Low** | **68%** | **68%** | **5%** | **0%** | **0%** | **59%** | **20%** | **0%** | **0%** | **0%** |
| **High** | **68%** | **68%** | **5%** | **0%** | **0%** | **59%** | **20%** | **0%** | **0%** | **0%** |
| **Immuno-Compromised** | **53%** | **53%** | **4%** | **0%** | **0%** | **0%** | **0%** | **0%** | **0%** | **0%** |

**All-Cause Pneumonia**

**PPV23.** Effectiveness of PPV23 against all-cause pneumonia was assumed to be zero, based on various published sources, and consistent with assumptions employed in a number of published economic studies (Table 9, Table 10) (Kraicer-Melamed 2016a, Kraicer-Melamed 2016b, Schiffner-Rohe 2016, Cho 2013, Moberley 2013, Smith 2013, Boccalini 2013, Kuhlmann 2012, Smith 2008, Evers 2007, Johnstone 2007, Sisk 2003, Fry 2002, Ortqvist 1998, Simberkoff 1986).

**PCV13.** Effectiveness of PCV13 against all-cause pneumonia—irrespective of setting of care—for immunocompetent persons was derived based on the:

- Estimated effectiveness of PCV13 against vaccine-type nonbacteremic pneumococcal pneumonia in the CAPiTA study (45%); and
- Percentage of all-cause pneumonia that is attributable to the serotypes in PCV13 (8%, in year 1 of modeling horizon) (McNeil 2014).

Estimated effectiveness of PCV13 against all-cause pneumonia (45% x 8% = 3.6%, in year 1 of the modeling horizon) was “anchored” to persons aged 73 years (mean age of study subjects in CAPiTA), and was assumed to persist at this level for the initial 5 years of the modeling horizon for the reasons described above. The rate of change in PCV13 effectiveness with age (i.e., for persons younger and older than 73 years of age) was equal to 50% of PPV23 values for IPD, and the rate of decline in PCV13 effectiveness over time (i.e., after the initial 5-year period) was assumed equal to 50% of PPV23 values for IPD beginning with the first year following receipt.

For immunocompromised adults, PCV13 effectiveness against all-cause pneumonia was assumed to be 65% of corresponding values for immunocompetent persons based on a trial of pneumococcal vaccination in children with and without HIV (Klugman 2003). Effectiveness estimates for immunocompromised persons employed in the model are consistent with data from French et al. on the effectiveness of PCV7 in HIV-infected adults (French 2010). Effectiveness of PCV13 was assumed to be the same irrespective of prior vaccination experience with PPV23 or PCV13.

**Suppl** Table 9. Effectiveness against all-cause pneumonia in the first year following vaccination

|  | **PCV13** | | | **PPV23** | | |
| --- | --- | --- | --- | --- | --- | --- |
|  | **Risk Profile** | | | **Risk Profile** | | |
| **Age Group** | **Low** | **High** | **Immuno-**  **Compromised** | **Low** | **High** | **Immuno-**  **Compromised** |
| **18 – 49** | **4.0%** | **4.0%** | **2.6%** | **0%** | **0%** | **0%** |
| **50 – 64** | **4.0%** | **4.0%** | **2.6%** | **0%** | **0%** | **0%** |
| **65 – 74** | **3.7%** | **3.7%** | **2.4%** | **0%** | **0%** | **0%** |
| **75 – 84** | **3.5%** | **3.5%** | **2.3%** | **0%** | **0%** | **0%** |
| **85 – 99** | **3.2%** | **3.2%** | **2.1%** | **0%** | **0%** | **0%** |

**Suppl Table 10. Effectiveness against all-cause pneumonia over time**

| **Age/ Risk** | **PCV13** | | | | | **PPV23** | | | | |
| --- | --- | --- | --- | --- | --- | --- | --- | --- | --- | --- |
|  | **Years Since Receipt of Vaccine** | | | | | **Years Since Receipt of Vaccine** | | | | |
|  | **1** | **5** | **10** | **15** | **20** | **1** | **5** | **10** | **15** | **20** |
| **18 – 49** |  |  |  |  |  |  |  |  |  |  |
| **Low** | **4.0%** | **4.0%** | **3.0%** | **1.5%** | **0%** | **0%** | **0%** | **0%** | **0%** | **0%** |
| **High** | **4.0%** | **4.0%** | **3.0%** | **1.5%** | **0%** | **0%** | **0%** | **0%** | **0%** | **0%** |
| **Immuno-**  **Compromised** | **2.6%** | **2.6%** | **1.9%** | **1.0%** | **0%** | **0%** | **0%** | **0%** | **0%** | **0%** |
| **50 – 64** |  |  |  |  |  |  |  |  |  |  |
| **Low** | **4.0%** | **4.0%** | **3.0%** | **1.5%** | **0%** | **0%** | **0%** | **0%** | **0%** | **0%** |
| **High** | **4.0%** | **4.0%** | **3.0%** | **1.5%** | **0%** | **0%** | **0%** | **0%** | **0%** | **0%** |
| **Immuno-**  **Compromised** | **2.6%** | **2.6%** | **1.9%** | **1.0%** | **0%** | **0%** | **0%** | **0%** | **0%** | **0%** |
| **65 – 74** |  |  |  |  |  |  |  |  |  |  |
| **Low** | **3.7%** | **3.7%** | **2.5%** | **1.1%** | **0%** | **0%** | **0%** | **0%** | **0%** | **0%** |
| **High** | **3.7%** | **3.7%** | **2.5%** | **1.1%** | **0%** | **0%** | **0%** | **0%** | **0%** | **0%** |
| **Immuno-**  **Compromised** | **2.4%** | **2.4%** | **1.6%** | **0.7%** | **0%** | **0%** | **0%** | **0%** | **0%** | **0%** |
| **75 – 84** |  |  |  |  |  |  |  |  |  |  |
| **Low** | **3.5%** | **3.5%** | **2.0%** | **0.7%** | **0%** | **0%** | **0%** | **0%** | **0%** | **0%** |
| **High** | **3.5%** | **3.5%** | **2.0%** | **0.7%** | **0%** | **0%** | **0%** | **0%** | **0%** | **0%** |
| **Immuno-**  **Compromised** | **2.3%** | **2.3%** | **1.3%** | **0.5%** | **0%** | **0%** | **0%** | **0%** | **0%** | **0%** |
| **85 – 99** |  |  |  |  |  |  |  |  |  |  |
| **Low** | **3.2%** | **3.2%** | **1.4%** | **0.1%** | **0%** | **0%** | **0%** | **0%** | **0%** | **0%** |
| **High** | **3.2%** | **3.2%** | **1.4%** | **0.1%** | **0%** | **0%** | **0%** | **0%** | **0%** | **0%** |
| **Immuno-**  **Compromised** | **2.1%** | **2.1%** | **0.9%** | **0.0%** | **0%** | **0%** | **0%** | **0%** | **0%** | **0%** |

**Medical-Care Costs**

Estimates of the cost of inpatient care for bacteremia, meningitis, and all-cause pneumonia were based on data from the Canadian Institute for Health Information (CIHI) and the Ontario Case Costing Initiative Database (Table 11, Table 12) (data on file). For all-cause pneumonia, the cost of hospitalization was assumed to be the same irrespective of the causative pathogen (i.e., for pneumococcal and non-pneumococcal pneumonia). While the assumed unit costs are higher than those for all-cause pneumonia reported elsewhere, they are largely consistent with estimates for pneumococcal pneumonia from other sources (Ontario Case Costing Initiative Database), which was deemed to be appropriate given the focus of this evaluation (i.e., on the prevention of pneumococcal pneumonia). Medical costs of all-cause pneumonia requiring outpatient care only were based on the study by Morrow et al., adjusted for inflation (Morrow 2007).

**Suppl Table 11. Medical-**care costs for IPD (per case)

|  | **Bacteremia** | | | **Meningitis** | | |
| --- | --- | --- | --- | --- | --- | --- |
|  | **Risk Profile** | | | **Risk Profile** | | |
| **Age Group** | **Low** | **High** | **Immuno-**  **Compromised** | **Low** | **High** | **Immuno-**  **Compromised** |
| **18 – 49** | **$44,747** | **$54,174** | **$55,061** | **$25,604** | **$30,998** | **$31,505** |
| **50 – 64** | **$44,627** | **$50,514** | **$58,934** | **$25,535** | **$28,904** | **$33,721** |
| **65 – 74** | **$33,023** | **$44,980** | **$46,954** | **$22,960** | **$31,273** | **$32,645** |
| **75 – 84** | **$35,465** | **$41,857** | **$46,355** | **$24,658** | **$29,101** | **$32,229** |
| **85 – 99** | **$33,487** | **$45,442** | **$42,628** | **$23,282** | **$31,594** | **$29,638** |

**Suppl Table 12. Medical-**care costs for all-cause pneumonia (per case)

|  | **Cases Requiring**  **Inpatient Care** | | | **Cases Requiring**  **Outpatient Care Only** | | |
| --- | --- | --- | --- | --- | --- | --- |
|  | **Risk Profile** | | | **Risk Profile** | | |
| **Age Group** | **Low** | **High** | **Immuno-**  **Compromised** | **Low** | **High** | **Immuno-**  **Compromised** |
| **18 – 49** | **$14,605** | **$16,556** | **$21,403** | **$96** | **$98** | **$113** |
| **50 – 64** | **$14,098** | **$16,001** | **$21,845** | **$95** | **$97** | **$112** |
| **65 – 74** | **$17,347** | **$19,750** | **$25,591** | **$95** | **$96** | **$112** |
| **75 – 84** | **$16,555** | **$20,234** | **$24,665** | **$94** | **$96** | **$111** |
| **85 – 99** | **$16,691** | **$20,537** | **$23,294** | **$94** | **$96** | **$111** |

**Utilities**

Age-specific utility values were obtained from the study by Sisk et al (2003). Disutilities from pneumococcal disease (i.e., IPD and all-cause pneumonia) were obtained from the study by Rubin et al. (2010), which employed data from Melegaro (2004).

**Vaccine Price**

The price used for PCV13 was the Pfizer confidential contract price. The price of PPV23 was set at $11.00 per dose, which was based on 2016 IMS data and an assumed discount to approximate the confidential government contract price. Vaccine administration cost ($15.59) was based on published data (Skowronski 2006).

**Cost-Effectiveness of Alternative Strategies for Use of**

**13-Valent Pneumococcal Conjugate Vaccine (PCV13) in Canadian Adults:**

**Online Supplement -- Listing of Measures, Parameters, and Sources/Assumptions**
